# The Apelinergic System in Kidney Disease: Novel Perspectives

**DOI:** 10.3390/ijms27010111

**Published:** 2025-12-22

**Authors:** Sara Saladich-Cavallé, Sara Núñez-Delgado, Linhui Huo, Frederic Pons-Pellicer, Irene Martínez-Díaz, Conxita Jacobs-Cachá, Sheila Bermejo, Jordi Vilardell-Vilà, Maria José Soler

**Affiliations:** 1 Nephrology and Transplantation Research Group, Vall d’Hebrón Research Institute (VHIR), Vall d’Hebron University Hospital, Vall d’Hebron Barcelona Hospital Campus, 08035 Barcelona, Spain; sarasaladich@gmail.com (S.S.-C.); sara.nunez@vallhebron.cat (S.N.-D.); linhui.huo@vhir.org (L.H.); frederic.pons@vhir.org (F.P.-P.); irene.martinez.diaz@vhir.org (I.M.-D.); sheila.bermejo@vallhebron.cat (S.B.); 2 Nephrology Department, Vall d’Hebron University Hospital, Vall d’Hebron Barcelona Hospital Campus, 08035 Barcelona, Spain; 3 Clinical Biochemistry, Drug Delivery and Therapy Research Group, Vall d’Hebrón Research Institute (VHIR), Vall d’Hebron University Hospital, Vall d’Hebron Barcelona Hospital Campus, 08035 Barcelona, Spain; conxita.jacobs@vhir.org

**Keywords:** apelin, APLNR, chronic kidney disease (CKD), CKD in persons with diabetes, renin–angiotensin–aldosterone system (RAS)

## Abstract

Chronic kidney disease (CKD) is a major complication of diabetes, affecting approximately 30–40% of patients. In many cases, CKD progresses to end-stage renal disease (ESRD). The peptide apelin and its receptor, APLNR, which is expressed in the endothelial cells of renal blood vessels, play a key role in glucose uptake and the regulation of vasodilation in the afferent and efferent glomerular arterioles. Numerous studies have demonstrated that the apelinergic system is dysregulated in various pathologies, including CKD in people with diabetes. In recent years, the apelinergic system has emerged as a promising therapeutic target for several diseases, with various apelin analogs and inhibitors being developed. In this review, we summarize the most recent literature on apelin and its cellular mechanisms of action, highlighting the role of the apelinergic system in various pathologies and its impact on patients with CKD and diabetes. Additionally, we explore the currently available analogs and inhibitors and discuss their potential therapeutic applications.

## 1. Introduction

Chronic kidney disease (CKD) is defined as a long-term loss of kidney function characterized by a glomerular filtration rate (GFR) < 60 mL/min/1.73 m^2^, a urinary albumin-to-creatinine ratio (UACR) ≥ 30 mg/g, kidney transplantation, non-urological hematuria, or structural renal abnormalities identified through histology or imaging (RICORS [1]). CKD has been declared a global health priority by the World Health Organization (WHO). It affects nearly 850 million people worldwide, and around 1.2 million deaths were attributed to CKD in 2017 [2].

Diabetes mellitus (DM) is a harmful chronic disease that causes damage to various organs in humans. Chronic kidney disease (CKD) is a major microvascular complication of DM. CKD affects around 40% and 30% of patients with type 1 and type 2 diabetes, respectively [3]. When CKD develops into end-stage renal disease (ESRD) in people with diabetes, the annual death rate exceeds 12%, compared to only 1% at early stages of DKD with normoalbuminuria [4]. According to research with data from 142 countries [5], the percentage of ESRD in persons with DM increased from 22.1% in 2000 to 31.3% in 2015. As a result of growing DM, the number of ESRD cases is predicted to reach 642 million by 2040 [6]. CKD poses a significant global health challenge, adversely affecting patient outcomes and imposing a substantial economic burden on healthcare systems worldwide. Glomerular endothelial cells (GECs) play a pivotal role in maintaining normal kidney function [7]. Not only do they function as a physical barrier to block abnormally large particles [8], but they also regulate blood flow and balance local pro- and anti-inflammatory mediators [9,10]. GECs are sensitive to blood sugar levels [7]. In DKD, GECs undergo a series of structural changes [6], which cause vascular and microenvironmental disturbances [10], leading to a decline in kidney function. Traditionally, type 2 diabetes management has focused on controlling blood glucose levels through lifestyle changes (diet, exercise, and weight loss), as well as through the use of various commonly prescribed drugs. Among the oral agents used as first-line therapy, metformin is particularly noteworthy for enhancing insulin sensitivity. Other existing medications act through alternative pathways, such as increasing insulin release by the pancreas (sulfonylureas or GLP-1 receptor agonists), increasing the body’s sensitivity to insulin (thiazolidinediones), or promoting the elimination of glucose in urine (SGLT2 inhibitors). Finally, when these drugs are not able to maintain blood glucose levels, treatment with insulin must be initiated [11]. It is important to note that classical hypoglycemic drugs, such as sulfonylureas or insulin, lack a renoprotective effect. Since sodium–glucose cotransporter 2 inhibitors (SGLT2i) and glucagon-like peptide-1 receptor agonists (GLP1A) have been reported to provide additional renoprotective effects besides blood sugar control, these new drugs are promising for treating CKD in people with diabetes [12,13,14].

Apelin is an endogenous ligand of a G protein-coupled receptor known as APLNR [15]. After its production, the 77-amino acid apelin preprotein is cleaved into shorter bioactive fragments, including apelin-12, apelin-13, apelin-17, and apelin-36 [16]. Apelin and APLNR are found in several tissues but are highly expressed in endothelial cells, intrarenal arteries, and renal arterioles [17], playing an important role in regulating endothelial glucose uptake, enhancing insulin sensitivity [18], and modulating vasodilation of the afferent and efferent glomerular arterioles [19].

Several studies in patients with CKD and diabetes have reported disturbances in apelin function. Habchi et al. found that patients with DM had significantly higher serum apelin levels compared to non-DM controls [20]. Another study found that patients with DM and higher apelin levels were at a higher risk of hospitalization, kidney function decline, and mortality [21]. One Chinese study reported a strong relationship between apelin gene single nucleotide polymorphisms and type 2 diabetes [22]. Furthermore, APLNR was found to colocalize with the sodium–glucose cotransporter 2 [17], indicating its potential role in renal glucose handling. Based on this evidence, APLNR agonists with improved pharmaceutical properties have been developed for potential future clinical use in medical treatment [23,24]. Although there is a lack of evidence in prospective studies on humans, the therapeutic effects of apelin have been tested using several diabetic animal models. In a type 1 diabetes mouse model, apelin-13 treatment reduced glomerular hypertrophy and mesangial matrix deposition, decreased albuminuria, and delayed renal inflammation [25]. In diabetic nephropathy (DN) rat models, apelin-13 administration not only decreased blood sugar levels but also improved kidney function and reduced renal fibrosis-related proteins [26]. These protective effects have been ascribed to increased endothelial nitric oxide synthase [26] and counteracting angiotensin II signals [26,27]. However, intraperitoneal injection of apelin-13 in KkAy mice (a polygenic model for human T2D) with D increased urinary albumin–creatinine and decreased creatinine clearance ratio [28], indicating deteriorated renal function. This harmful effect was caused by the inhibition of podocyte autophagy through several pathways and was reversed by the apelin antagonist F13A [28].

All of the aforementioned studies highlight the complex role of apelin in diabetes and CKD. In this review, we focus on reviewing the function of apelin in both normal and disease conditions. We also discuss its potential benefit as a new therapeutic target.

## 2. The Apelinergic System

Apelin belongs to the adipokine family and was first identified by Tatemoto et al. in 1998 [15]. In humans, the *APLN* gene is located on the long arm of the X chromosome (Xq25-26.1) and encodes a 77-amino acid precursor proprotein known as pre-proapelin. Cleavage of the last 22 amino acids of the N-terminal region generates proapelin-55, which can be processed into several shorter active isoforms, including apelin-36, apelin-17, and apelin-13. All of these active forms have the same 12 C-terminal residues in common, which are essential for binding to their receptor [29].

**Figure 1:**
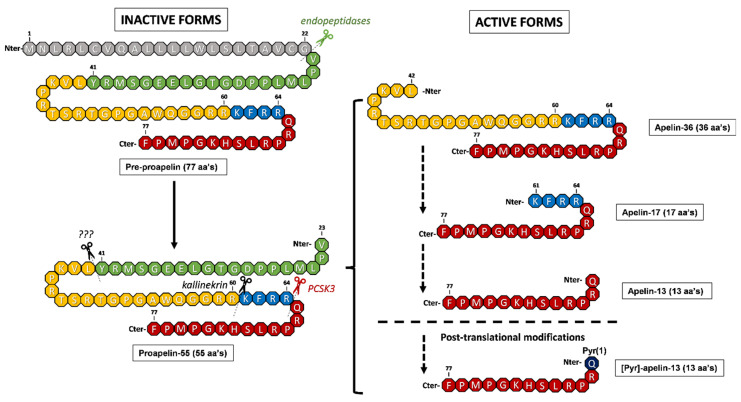
Processing and generation of apelin peptides. Pre-proapelin (77 aa’s) is cleaved by different endopeptidases (green), which eliminates the N-terminal domain (1–22) and produces proapelin (55 aa’s). The rest of the active forms are generated by different enzymes, such as PCSK3/furin (red) or kallinekrin (yellow), but some of them remain to be discovered (???). All active apelin isoforms can be generated by a sequential processing of the isoforms (traditional model) (dashed arrows) or directly from proapelin-55 (recent model). [Pyr]-apelin-13 is formed through the cyclisation of the N-terminal glutamine of apelin-13.

The presence of these 12 C-terminal residues allows all the peptides derived from proapelin-55 to be biologically active. In fact, although proapelin-55 is considered as an inactive proprotein, its C-terminal domain gives it some biological activity [29]. While all active apelin isoforms exhibit variable biological activity, they have short half-lives of less than 5 min due to rapid degradation by proteases [30]. The presence of different cleavage sites for several proteases along the apelin sequence has led to debate about the exact mechanism by which the plethora of active apelin isoforms is obtained. While it has been established that the conversion of pre-proapelin-77 to proapelin-55 occurs through the action of uncharacterized endopeptidases that cleave between residues Gly32 and Gly33, the conversion of apelin-55 to the remaining isoforms is still unknown. While the traditional model suggests that all the isoforms derived from proapelin-55 arise through sequential cleavage by different proteases, a recent model suggests that all apelin isoforms can also be generated directly from the precursor proapelin-55 (Figure 1).

Several enzymes are responsible for this cleavage. Notably, proprotein convertase subtilisin/kexin type 3 (PCSK3), also known as furin, cleaves residues Arg64 and Gln65 to produce apelin-13. Spontaneous cyclization of the N-terminal glutamine in apelin-13 forms pyroglutamate ([Pyr]-apelin-13), enhancing its resistance to enzymatic degradation and extending its biological activity [31] (Figure 1). Additional proteases contribute to the inactivation of apelin: angiotensin-converting enzyme 2 (ACE2), which acts at the C-terminal phenylalanine residue; neprilysin (neutral endopeptidase), which targets the “RPRL” region (Arg2-Leu5 and Leu5-Ser6); and plasma kallikrein (KLKB1), which cleaves apelin-17 at the first three N-terminal residues, resulting in the production of apelin-14 [32].

The measurement of apelin peptide levels in plasma remains challenging for two main reasons: (i) the conserved region of the 17 C-terminal amino acid residues shared by all apelin peptides and (ii) their short half-life. Currently, plasma apelin levels are mainly determined using commercial kits based on enzyme immunoassays. As these kits use antibodies against the C-terminal domain, they cannot distinguish between different peptide isoforms [33]. More complex methods, such as high-performance liquid chromatography–radioimmunoassay (HPLC-RIA), can partially solve the problem by allowing the identification of a limited number of isoforms [33]. Nevertheless, to our knowledge, methods based on mass spectrometry (HPLC-MS/MS) still lack sufficient reproducibility [17,34]. Furthermore, the rapid clearance of apelin peptides further complicates the study of the pharmacokinetic and pharmacodynamic properties of different isoforms [17,35].

Apelin and its various isoforms, which contain C-terminal fragments of variable lengths, are endogenous ligands for APLNR, a G protein-coupled receptor (GPCR) of class A. The receptor was identified by O’Dowd et al. as an orphan G-coupled receptor in 1993 [36]. Consisting of 380 amino acids, the receptor includes seven hydrophobic transmembrane domains. APLNR activation by apelin peptides engages G proteins (Gα, Gβ, Gγ), thereby triggering multiple signaling pathways. Gαi-mediated inhibition of cAMP production, alongside phosphatidylinositol 3-kinase (PI3K)/protein kinase B (AKT) activation, induces autophagy and promotes p70S6 kinase phosphorylation via mechanistic target of rapamycin (mTOR). Simultaneously, extracellular signal-regulated kinase (ERK1/2) activation also contributes to p70S6 kinase phosphorylation, collectively promoting cell proliferation and inhibiting apoptosis [37]. Additionally, Akt and AMPK (AMP-activated protein kinase) activation stimulate endothelial nitric oxide synthase (eNOS) phosphorylation, increasing nitric oxide (NO) release from endothelial cells and inducing vasodilation. APLNR signaling can also be mediated by β-arrestin-2, which regulates receptor internalization and desensitization.

In humans, apelin mRNA is expressed throughout the vasculature and the central nervous system, as well as in many other organs, including the heart, lungs, and kidneys. However, it is predominantly found in endocardial and vascular endothelial cells, suggesting that circulating apelin originates from these tissues [38]. The different apelin isoforms vary not only in their tissue distribution but also in their biological potency. Apelin-13 and its pyroglutamated form, [Pyr1]-apelin-13, exhibit greater potency in activating the receptor compared to apelin-36. The shorter isoforms are especially prevalent in cardiac tissue, whereas apelin-36 is more abundant in the lungs, testes, and uterus. In plasma, apelin-17 and [Pyr1]-apelin-13 are considered as the predominant isoforms [39].

Functionally, the apelin/APLNR axis plays a central role in cardiovascular and metabolic systems regulation. In the vascular system, apelin induces vasodilation, reduces blood pressure, and promotes angiogenesis. In the kidneys, apelin enhances renal blood flow, increases diuresis, and exerts anti-inflammatory and antifibrotic effects. Apelin also contributes to fluid homeostasis by inhibiting vasopressin release from the hypothalamus, thereby suppressing water intake. Moreover, it promotes brown adipose tissue formation and mitochondrial biogenesis in white adipose tissue, improves glucose metabolism in skeletal muscle, and enhances insulin sensitivity [27].

Despite sharing approximately 40% sequence homology with the angiotensin II (AngII) receptor, APLNR does not interact with AngII due to its specific recognition of the conserved structural motifs of apelin-derived peptides [40]. As APLNR is a class A G protein-coupled receptor (GPCR), it can form both homo- and heterodimers through interactions within its transmembrane domains [41]. Interestingly, although heterodimerization between APLNR and the AngII receptor has not been demonstrated, APLNR can form heterodimers with other GPCRs, such as the A_2_A adenosine receptor, the D_2_ dopamine receptor [42], the neurotensin-1 receptor [43], and the κ-opioid receptor [44].

**Figure 2:**
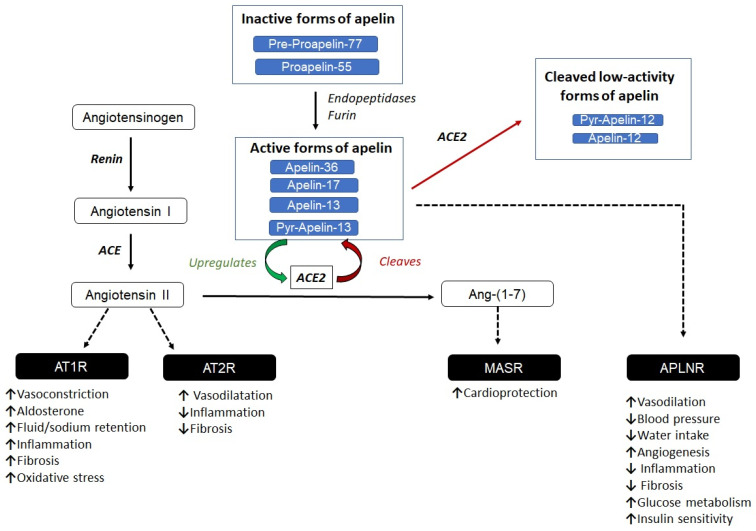
Interrelation between the renin–angiotensin system (RAS) and the apelin system. In the RAS system, angiotensinogen is converted to angiotensin I by the action of renin, which is then transformed to angiotensin II by ACE. ACE2 can convert angiotensin II to Ang-(1-7), triggering opposite effects in the renal system. The active forms of apelin interact with APLNR, activating different biological responses. The active forms apelin-13 and Pyr-apelin-13 upregulate ACE2 expression. However, ACE2 cleaves the C-terminal phenylalanine of apelin peptides (apelin-13 and Pyr-apelin-13), producing low-activity forms of apelin (apelin-12 and Pyr-apelin-12). This generates a negative feedback loop that connects the apelin system with the RAS pathway. **Dashed lines**: Receptor interaction.

Nevertheless, a significant point of interaction exists between the apelinergic system and the renin–angiotensin–aldosterone system (RAS) through their common enzymatic regulator, angiotensin-converting enzyme 2 (ACE2). While ACE2 cleaves angiotensin II into the vasodilatory fragment angiotensin 1–7, it also degrades apelin peptides, particularly at the C-terminal phenylalanine residue. Apelin, in turn, has been shown to upregulate ACE2 expression, which suppresses angiotensin II signaling via AT1R, favoring the vasoprotective angiotensin 1–7–Mas receptor axis. Through this mechanism, the apelin/APLNR system not only counterbalances the hypertensive effects of angiotensin II but also contributes to the fine-tuning of vascular tone and systemic blood pressure [45] (Figure 2).

## 3. Role of Apelin in Diseases

As previously mentioned, apelin-13 is a potent endogenous ligand for APLNR, which is coupled to G proteins [15] and is known to participate in a wide range of physiological processes, such as cardiovascular regulation, fluid homeostasis, and metabolic balance [46]. Apelin behaves like a hormone; its expression has multiple effects on its target tissues [15]. Its role in various physiological processes is mainly attributed to its antioxidant, anti-inflammatory, and antiapoptotic properties [47]. In addition, the Apelin/APLNR axis is widely distributed among different cell types, in such a way that any alteration of this system would lead to imbalances in tissue homeostasis or even several pathologies, depending on the affected tissue. Due to its properties and its involvement in various physiological processes, the role of the apelin/APLNR axis has been studied in several biological processes and systemic diseases, some of which are detailed below.

### 3.1. The Role of Apelin in Inflammatory and Fibrotic Processes

Apelin has been observed to affect inflammatory and fibrotic responses and is involved in diseases where these processes are deregulated. Apelin inhibits the expression of molecules that are involved in inflammatory diseases, such as lipoprotein lipase (LPL), which promotes lipid accumulation and the secretion of pro-inflammatory cytokines in macrophages, thereby exacerbating the pathological process. Zhang et al. showed that apelin decreased LPL expression by activating the APJ/PKCα/miR-361-5p signaling pathway in THP-1 macrophage-derived squamous cells. This led to decreased secretion of pro-inflammatory cytokines and lipid accumulation. In addition, apelin has also been shown to reduce the expression of adhesion molecules, such as intercellular cell adhesion molecule-1 (ICAM-1) and E-selectin [48], which facilitate the accumulation of macrophages in an inflamed area. Therefore, a decrease in the expression of both ICAM-1 and E-selectin will improve the prognosis of the disease. It is important to note that this inflammatory process is affected by oxidative stress, and apelin appears to play a significant role in this context. In fact, apelin can reduce the production of reactive oxygen species (ROS) by inhibiting NADPH oxidase and upregulating the expression of superoxide dismutase (SOD) and catalase. In diabetic nephropathy, apelin signaling suppresses oxidative stress, inflammation, and fibrosis, thereby improving mitochondrial function and restoring antioxidant enzyme expression [49,50,51].

### 3.2. Apelin and Cancer: Role in Angiogenic Processes

Additionally, apelin has been shown to have potent proangiogenic properties [52], inducing vessel formation and having detrimental effects in patients with cancer by facilitating tumor progression and metastatic processes [53,54]. For this reason, inhibitors of the apelin/APLNR axis are currently being investigated as potential antitumoral agents with the ultimate goal of avoiding tumor vascularization [55]. Other studies, however, suggest that there is no direct relationship between the apelin/APLNR axis and the degree of tumor malignancy; rather, it depends on the cell type that generates the tumor [56].

### 3.3. Apelin and the Nervous System: Role in Neural Damage

Apelin also appears to be involved in neural system damage due to its antiapoptotic properties. In vitro assays performed in an oxygen–glucose deprivation model of PC12 human cells (in order to simulate spinal cord injury (SCI)) and in Sprague Dawley rats with induced SCI (by T9 lateral hemisection) have shown that apelin-13 has a protective effect by reinforcing autophagy and attenuating apoptosis [57]. Furthermore, apelin-13 has also been found to have a regenerative effect by enhancing SCI repair through macrophage regulation and M1/M2 polarization, as well as preventing apoptosis and promoting autophagy [58]. These results suggest that apelin may be involved in inflammatory and reparative/fibrotic processes in several tissues.

### 3.4. The Role of Apelin in Cardiovascular Diseases

In the cardiovascular system, apelin acts on the endothelial cells of blood vessels and has positive inotropic and vasodilator effects [59]. By promoting nitric oxide (NO)-dependent vasodilation, it may improve outcomes in heart failure [60]. In fact, the potential therapeutic benefits of apelin on myocardial function have been demonstrated in dogs [61]. In concordance with these results, APLNR receptor agonists have been shown to induce a sustained enhancement in cardiac output in heart disease through the activation of the apelin/APLNR axis [62].

Regarding its role in atherosclerosis, apelin has been demonstrated to exert a protective effect by acting on several pathways. One of these pathways, as mentioned above, involves the production of NO, promoting vasodilation while blocking the vasoconstrictor properties of angiotensin II [63].

### 3.5. The Role of Apelin in Hypertension and CKD

Arterial hypertension (AH) is often a consequence of cardiovascular and kidney damage. Kidney damage caused by hypertension contributes to the progression of CKD and is closely related to AH, acting as a factor in raising blood pressure levels. Consequently, declining kidney function is often associated with increased blood pressure [64].

In AH animal models, such as Rattus Norvergicus Wistar albino rats, it was observed that APLNR was lower in the kidney than in healthy rat models. Conversely, elevated expression of APLNR was detected in the cardiac tissue of hypertensive animals [65]. Indeed, a study conducted on two-kidney-one-clip hypertensive rats (2K1C) found that the infusion of 40 μg/Kg apelin caused a substantial drop in both systolic and diastolic blood pressure levels [66]. However, other studies conducted on normotensive Sprague Dawley rats treated with angiotensin II to induce hypertension showed that daily administration of apelin was unable to reduce the BP elevation caused by angiotensin II or the harmful effects, such as cardiac hypertrophy and fibrosis [67].

Is important to highlight that in a double-blind and placebo-controlled study of 12 patients with CKD (5 with IgA nephropathy, 2 with ANCA-associated vasculitis, 2 with hypertensive nephropathy, 1 with anti-glomerular basement membrane disease, 1 with reflux nephropathy, and 1 with chronic interstitial nephritis), as well as a control group of healthy patients, found that apelin reduced blood pressure and caused peripheral vasodilation in the CKD group. At the same time, apelin increased renal blood flow and reduced renal vascular resistance in these patients. Finally, a significant decline in the glomerular filtration rate and proteinuria was also detected in the CKD group treated with apelin [68]. Similarly, in a murine model of contrast-induced acute kidney injury, it was shown that apelin has a renoprotective effect. More specifically, studies in both murine models and human HK-2 renal cell lines showed that the administration of apelin-13 mitigates endoplasmic reticulum stress and the formation of reactive oxygen species in renal tubular cells.

Conversely, other studies carried out on patients with CKD and autosomal-dominant polycystic kidney disease showed that circulating serum apelin levels were increased in these patients and were directly related to the progression of renal dysfunction [29]. Taken together, these studies suggest that modulating apelin levels may exert a therapeutic potential in preventing contrast-media-induced acute kidney injury [47].

## 4. The Role of Apelin in Human Diabetes and CKD

Circulating apelin levels have been studied in people with diabetes. Case–control studies in children with type 1 diabetes have reported consistently higher apelin levels compared to healthy controls [69,70,71]. These levels appear to be even higher than those observed in adults with type 2 diabetes (T2D) [20] and remain elevated when compared to those of non-diabetic individuals [72,73]. Therefore, it appears that apelin levels increase in the presence of diabetes and CKD [73,74]. While some discrepancies were found [70], studies describing the relationship between apelin and albuminuria in patients with diabetes showed a positive correlation between the two parameters [71,72,75]. This relationship was also observed in the study by İçen et al., who compared 120 patients with T2D and different degrees of renal impairment. The study showed that apelin levels increased progressively with each stage of albuminuria. However, in the study, patients with high albuminuria (>300 mg/day) and an increase in creatinine had lower apelin levels than those with high albuminuria and no increase in creatinine [76]. Jiang et al. also demonstrated a negative correlation between apelin and creatinine in patients with cognitive impairment and mild kidney dysfunction [77]. Nevertheless, other observational studies have shown an increase in apelin levels with the increase in creatinine or a decrease in eGFR in mild to moderate kidney dysfunction [71,73,74]. A recent study by Özdemir et al. compared 88 patients with T2D and 32 healthy controls and found that patients with T2D had slightly lower levels of apelin-13. This suggests that there may be a relationship between apelin and T2D that goes beyond the pathophysiology of diabetes itself.

Higher levels of apelin were also found in patients with chronic kidney disease (CKD), with no significant difference observed between those with and without diabetic kidney disease (DKD) when kidney dysfunction was severe [78]. These discrepancies demonstrate the heterogeneity in these observational studies and emphasize the importance of evaluating the role of apelin at different stages of DKD.

The prognostic value of apelin in DKD was evaluated in a prospective observational study of 150 patients with type 2 diabetes and CKD at any stage. The mean creatinine level was 1.9 ± 1.0 mg/dL, and none of the patients had a history of CVD. Patients with higher apelin levels exhibited a higher eGFR, lower albuminuria, and better overall survival during a mean follow-up period of 35.7 months. Circulating apelin levels were independently associated with lower cardiovascular mortality (hazard ratio 0.981, 95% confidence interval (CI) 0.967–0.996, *p* = 0.012) and lower cardiovascular hospitalization (odds ratio 0.548, 95% CI 0.302–0.817, *p* = 0.004), regardless of kidney function parameters. However, in the study, apelin levels were not significantly associated with a reduction in the progression to end-stage kidney disease or the need for kidney replacement therapy in the population [21].

Other markers were also studied in relation to the pathophysiology of apelin in DKD. Apelin is negatively correlated with TGF-1β, one of the main mediators of fibrosis, in patients with DKD. This suggests that apelin plays a protective role against glomerular sclerosis [73]. Increased levels of interleukins, such as IL-6, as well as other inflammatory parameters, namely, oxLDL and the pro-inflammatory adipokines resistin and visfastin, have been observed in patients with T2D and lower levels of circulating apelin. This suggests that apelin plays a protective role against the inflammatory state present in patients with T2D and CKD [21]. However, the specific relationship between inflammation, apelin, and kidney disease in T2D does not seem to differ when compared to other causes of CKD [78]. In T1D patients with CKD, there is no association between apelin, nitric oxide metabolism abnormalities, and endothelial dysfunction [70]. Furthermore, anti-inflammatory adipokines, such as adiponectin, were found to be reduced in T2D patients with lower levels of apelin, but these results were inconsistent in T1D patients [21,69]. The fact that apelin increases alongside albuminuria could suggest that a direct worsening effect on podocyte permeability had a detrimental impact in advanced states of DKD [73]; however, controlled clinical studies are needed to establish this possible relationship.

The effect of specific diabetes treatments on apelin and DKD has not been directly studied, but insights into their potential role have emerged from studies of other pathologies. In a randomized study of patients with breast or colorectal cancer, who were assigned to receive either aerobic exercise, metformin, both treatments, or a placebo, metformin was found to decrease apelin levels during the follow-up period [79]. Two prospective studies on patients with T2D and heart failure found that treatment with dapagliflozin increased circulating apelin levels [80,81]. While none of these studies assessed the relationship between apelin and kidney parameters, these findings show promising results for the treatment of T2D, highlighting the potential of apelin to enhance insulin resistance and delay CKD progression.

In conclusion, according to the current evidence, the role of apelin in the pathophysiology and progression of DKD is complex. To date, no prospective studies have demonstrated a relationship between the apelinergic system, DKD pathophysiology, and progression. Furthermore, no histopathological studies have identified apelin in kidney biopsies from patients with T2D. Further research is needed to assess the potential of apelin as a biomarker of renal and cardiovascular progression in T2D patients and to investigate the interaction between pharmacological T2D treatments and renal function.

## 5. Apelin Analogs and Their Therapeutic Potential

The apelin system has emerged as a promising target for the treatment of cardiovascular diseases, metabolic disorders, and cancer, given its role in vasodilation, angiogenesis, glucose metabolism, and cell proliferation [82]. Therefore, numerous apelin analogs and inhibitors have been developed to modulate its activity; however, clinical studies are challenging, as apelin peptides have a short half-life (just a few minutes in humans) and APLNR rapidly desensitizes via coupling to β-arrestins [32]. Consequently, current efforts are focused on developing metabolically stable apelin analogs. The most common approaches applied in apelin research involve classic chemical substitution, cyclization, acylation, and PEGylation.

The cyclization of apelin was first reported in 2008 when Fukamizu’s research group generated three cyclic analogs of apelin-12 that were capable of inhibiting cAMP production and inducing ERK phosphorylation [32]. More recent work has focused on cyclic derivatives of apelin-13, resulting in the development of macrocyclic analogs with enhanced receptor affinity and an extended plasma half-life of over three hours. In rat heart ex vivo preparations using the Langendorff system, analogs such as compounds 13 and 15 exhibited potent inotropic effects at concentrations as low as 0.003 nM, which is an order of magnitude more efficacious than native apelin-13 [83]. Given that apelin-17 is susceptible to degradation by plasma kallikrein (KLKB1), which diminishes its hypotensive activity, modifications such as N-terminal palmitoylation or PEGylation have been applied to protect against proteolysis. Optimized PEGylated apelin-17 analogs have demonstrated significantly prolonged half-lives of up to 18 h, as well as enhanced hypotensive and cardioprotective effects in the same Langendorff heart assays [84].

Several APLNR agonist compounds have been designed to block its activation. The first agonist described was E339-3D6, which displayed nanomolar affinity and acted as a full agonist for receptor internalization. However, it was only partially capable of inhibiting forskolin-induced cAMP production. Next came ML-233, the first non-peptide agonist capable of activating both the G protein and β-arrestin pathways. However, the molecule demonstrated low potency (EC_50_ = 3.7 μM). A subsequent improvement was the development of CMF-019 by Sanofi, which is based on a substituted benzimidazole scaffold. CMF-019 demonstrated potent calcium mobilization activity (EC_50_ = 47 nM), low β-arrestin recruitment, minimal receptor internalization, and a favorable G protein-biased profile. This compound induced vasodilation, enhanced cardiac contractility, and conferred antiapoptotic effects in pulmonary artery endothelial cells without triggering receptor desensitization [85]. Similarly, MM07 emerged as another G protein-biased agonist with high binding affinity and superior cardiovascular benefits compared to native [Pyr1]-apelin-13 [86]. Later, Sanford-Burnham filed a follow-up patent for a pyrido-pyrimidin-4-one scaffold derivative that acts as a full agonist by inhibiting the cAMP pathway (EC_50_ = 6.7 nM). Efforts have also been directed towards the synthesis of pyrazole, hydroxypyrimidone, and triazole-based compounds, such as AMG986, produced by Ason and colleagues. Although AMG986 exhibited reduced cAMP inhibitory potency compared to [Pyr1]-apelin-13, it demonstrated enhanced recruitment of both G proteins and β-arrestin. In a murine model of diastolic dysfunction, AMG986 also improved stroke volume and ejection fraction without significantly affecting blood pressure. This compound has progressed to a Phase I double-blind, placebo-controlled clinical trial (NCT03276728) to assess its pharmacokinetics and pharmacodynamics in healthy volunteers and patients with heart failure [27]. It has been demonstrated that BMS-986224 has physiological effects similar to those of [Pyr1]-apelin-13, and it has shown a promising therapeutic potential in preclinical models of heart failure. Like AMG 986, BMS-986224 was well tolerated in healthy humans and patients with heart failure. However, data regarding its clinical efficacy are inconsistent, necessitating additional studies to clarify its possible therapeutic potential [62] (Table 1).

In addition to the pursuit of APLNR agonists, the development of receptor antagonists has gained traction, particularly for their potential antiangiogenic applications in cancer [91]. Among the most well characterized is MM54, a cyclic peptide based on the RPRL motif that selectively inhibits β-arrestin-mediated receptor internalization. It has shown high specificity across a wide panel of GPCRs, including AT1R, as well as various ion channels [92]. In contrast, other antagonists like ALX40-4C and protamine, composed of polycationic sequences, display low affinity and limited selectivity for the apelin receptor (Table 2).

Recent clinical evidence supports the therapeutic potential of apelin analogs in the treatment of cardiovascular and renal diseases. A randomized, double-blind, placebo-controlled, crossover study evaluated the effects of [Pyr1]-apelin-13 in 24 subjects (12 patients with chronic kidney disease (CKD) and 12 matched healthy controls). Intravenous administration of [Pyr1]-apelin-13 at doses of 1 nmol/min and 30 nmol/min demonstrated significant cardiovascular and renal benefits. The peptide reduced systemic vascular resistance while increasing cardiac output and renal blood flow. Additionally, it enhanced natriuresis and free water clearance, indicating its potential role in regulating fluid balance. In CKD patients, [Pyr1]-apelin-13 also led to a significant reduction in proteinuria, further supporting its renoprotective effects [68].

Overall, the apelin system remains a promising therapeutic target. Apelin analogs have shown promise in the treatment of cardiovascular and metabolic diseases, whereas receptor antagonists may be valuable in oncology through their antiangiogenic effects. As apelin actively participates in tumor progression, it should be considered as a possible therapeutic target, especially in patients with cancer [98]. However, several studies have reported divergent effects of apelin on tumor progression, indicating that its impact could depend on the characteristics of the tumor microenvironment and the degree of associated immune infiltration [55,99,100,101]. It is worth mentioning that activation of the apelin system has different effects depending on the tissue microenvironment. For this reason, the development of new tissue-specific receptor agonists to reduce the possible risk of unwanted off-target effects could be of great interest. Ongoing preclinical and clinical efforts are therefore essential to refine the pharmacokinetic properties of these agents and to establish their long-term safety and efficacy in diverse pathological contexts.

## 6. Conclusions

The apelin system is dysregulated in several pathological conditions, including cancer, cardiovascular diseases, and CKD. The system has been clearly associated with RAS, inflammation, and fibrosis. Its involvement in different diseases has, in recent years, led to the synthesis of different analogs to apelin (like [Pyr]-apelin-13), as well as different antagonists for its receptor (such as MM54, protamine, or ML221), which are more stable and show a promising therapeutic potential. Despite considerable advances in our understanding of the apelin system, many aspects of its mechanism of action and involvement in several pathologies remain unknown. For this reason, we summarized the most recent insights regarding the role of apelin and its receptor in various diseases, mainly focusing on CKD.

## Figures and Tables

**Table 1:** Table of some APLNR agonists and their binding affinities.

Ligand	Action	Binding Affinity	Units	Effects	Reference
**CYCLO APELIN-12 (1–12)**	Full agonist	6.3	pEC_50_	Inhibit cAMP accumulation, increase Akt and ERK phosphorylation	Hamada et al., 2008 [87]
**COMPOUND 15/ANALOG 15**	Full agonist	0.15	K_i_	Activate Gαi1 and recruit β-arrestin2	Trân et al., 2018 [83]
**PEG-17A2**	Full agonist	6.3	EC_50_	Calcium release, lower blood pressure	Fischer et al., 2020 [84]
**E339-3D6**	Agonist	6.4	pK_i_	Vasorelaxation, reduce vasopressin release	Iturrioz et al., 2010 [88]
**ML-233**	Full agonist	3.7	EC_50_	Reduce cAMP, increase APJ internalization	Khan et al., 2011 [89]
**CMF-019**	Full agonist	8.58	pK_i_	Prevent apoptosis, reduce artery pressure, increase cardiac contractility	Read et al., 2021 [85]
**MM07**	Full agonist	9.5	pEC_50_	Increase cardiac output (rat) and forearm blood flow (human)	Brame et al., 2015 [86]
**AMG986**	Agonist	9.5	pEC_50_	Increase stroke volume, ejection fraction, and heart rate; decrease cardiac afterload	Ason et al., 2020 [90]
**BMS-986224**	Agonist	9.5	pKd	Increase stroke volume and cardiac output; decrease arterial pressure	Gargalovic et al., 2021 [62]

List of abbreviations: EC_50_ (50% Effective Concentration), K_i_ (inhibition constant), pEC_50_ (E_50_ negative logarithm), pK_i_ (K_i_ negative logarithm), pKd (Dissociation Constant Negative Logarithm).

**Table 2:** Table of some APLNR antagonists and their binding affinities.

Ligand	Action	Binding Affinity	Units	Effects	References
**MM54**	Antagonist	8.2	pK_i_	Inhibit cAMP accumulation	Macaluso et al., 2011 [93]
**ALX40-4C**	Antagonist	5.5	pIC_50_	Block cell membrane fusion	Zhou et al., 2003 [94]
**PROTAMINE**	Antagonist	6.4	pK_i_	Antagonize G-protein- and β-arrestin-dependent pathways	Le Gonidec, et al. [95]
**ML221**	Antagonist	0.70	pIC_50_	Inhibit cAMP production and β-arrestin recruitment	Maloney et al., 2012 [96]
**4-AMINOQUINOLINE**	Selective antagonist	0.556	pIC_50_	Suppress endothelial tube formation and neovascularization	McAnally et al., 2018 [97]

List of abbreviations: IC_50_ (inhibition constant of 50%), K_i_ (inhibition constant), pIC_50_ (IC_50_ negative logarithm), pK_i_ (K_i_ negative logarithm).

## Data Availability

No new data were created or analyzed in this study. Data sharing is not applicable to this article.
